# Sublay repair for primary superior lumbar hernia with the Kugel patch

**DOI:** 10.1111/ans.15866

**Published:** 2020-03-24

**Authors:** Ronggui Lin, Tianhong Teng, Xianchao Lin, Fengchun Lu, Yuanyuan Yang, Congfei Wang, Yanchang Chen, Heguang Huang

**Affiliations:** ^1^ Department of General Surgery Fujian Medical University Union Hospital Fuzhou People's Republic of China

**Keywords:** lumbar hernia, open repair

## Abstract

**Background:**

A superior lumbar hernia is a posterior ventral hernia that is rarely encountered in the clinical setting. However, no standard operative strategy exists for superior lumbar hernia repair at present.

**Methods:**

Twelve patients with primary superior lumbar hernia who underwent sublay repair via the retroperitoneal space with the Kugel patch between December 2008 and June 2019 were included in this study. The demographic, peri‐operative and post‐operative data of the patients were collected to analyse the effectiveness of this technique.

**Results:**

All patients underwent an uneventful operation. The median operative time was 60 min, and the median blood loss was 35 mL. The median hernia defect area was 16 cm^2^. Five medium‐sized Kugel patches (11 cm × 14 cm) and seven large‐sized Kugel patches (14 cm × 17 cm) were used for the repairs. The median visual analogue scale score on post‐operative day 1 was 3. The median time to removal of drainage was 3 days. The median duration of the hospital stay was 3 days. No serious post‐operative complications, including seroma, haematoma, incision or mesh infection, recurrence and chronic pain, occurred during the follow‐up period.

**Conclusion:**

Sublay repair for primary superior lumbar hernia with the Kugel patch shows benefits including a reliable repair, minimal invasiveness and few post‐operative complications.

## Graphical abstract

Repair for superior lumbar hernias is technically challenging. Sublay repair using the Kugel mesh is a feasible and effective method for superior lumbar hernias. Sublay repair for superior lumbar hernias provides the advantages of reliable repair and rapid recovery.

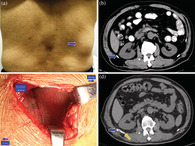

## Introduction

A lumbar hernia is defined as the abdominal organs or retroperitoneal fat protruding although defect areas in the lumbar region. Lumbar hernia is a rarely encountered posterior ventral hernia,^1^ which accounts for <2% of all external abdominal hernias. Lumbar hernia is divided into congenital lumbar hernia (20%) and acquired lumbar hernia (80%) according to the aetiology.^2^ Acquired lumbar hernia is subdivided into primary and secondary lumbar hernias, and the former accounts for approximately 55% of these hernias.^3^ Primary lumbar hernia consists of superior and inferior lumbar hernias, which protrude through two possible defect areas, the superior and inferior lumbar triangles. The superior lumbar triangle has a larger defect area and a weaker floor than the inferior lumbar triangle and contributes to more superior lumbar hernias in the clinical setting.^4^

Superior lumbar hernia, also called Grynfeltt hernia, refers to a hernia that protrudes through the superior lumbar triangle in the lumbar region.^5^ The superior lumbar triangle (Grynfeltt's triangle) is superiorly bound by the 12th rib and inferior posterior serratus muscle, medially bound by the erector spinae muscle, and laterally bound by the internal oblique muscle. The roof of Grynfeltt's triangle is formed by the latissimus dorsi muscle, while the floor is formed by the aponeurosis of the transversalis muscle.^6^ A superior lumbar hernia often manifests as a flank, reducible bulge beneath the 12th rib, and the defect area is usually detected by physical and imageological examinations. However, a few patients were misdiagnosed as having lipoma^7^ or were initially misdiagnosed with a stomach ache or constipation symptoms.

Surgical repair is suggested for primary superior lumbar hernias to reduce the potential risk of incarceration and strangulation.^8^, ^9^ However, same with other marginal external hernias, superior lumbar hernias are difficult to repair.^10^ Due to the extremely low morbidity, most reports of superior lumbar hernia are case reports or retrospective case studies with only a few patients enrolled.^11^, ^12^, ^13^ Accordingly, many controversies regarding superior lumbar hernia repair exist due to the lack of large prospective case–control studies.

In the present study, we performed sublay repair for 12 patients with superior lumbar hernia via the retroperitoneal space with the Kugel patch. No serious post‐operative complications occurred during the follow‐up period, indicating the safety and feasibility of the technique.

## Methods

**Figure 1:**
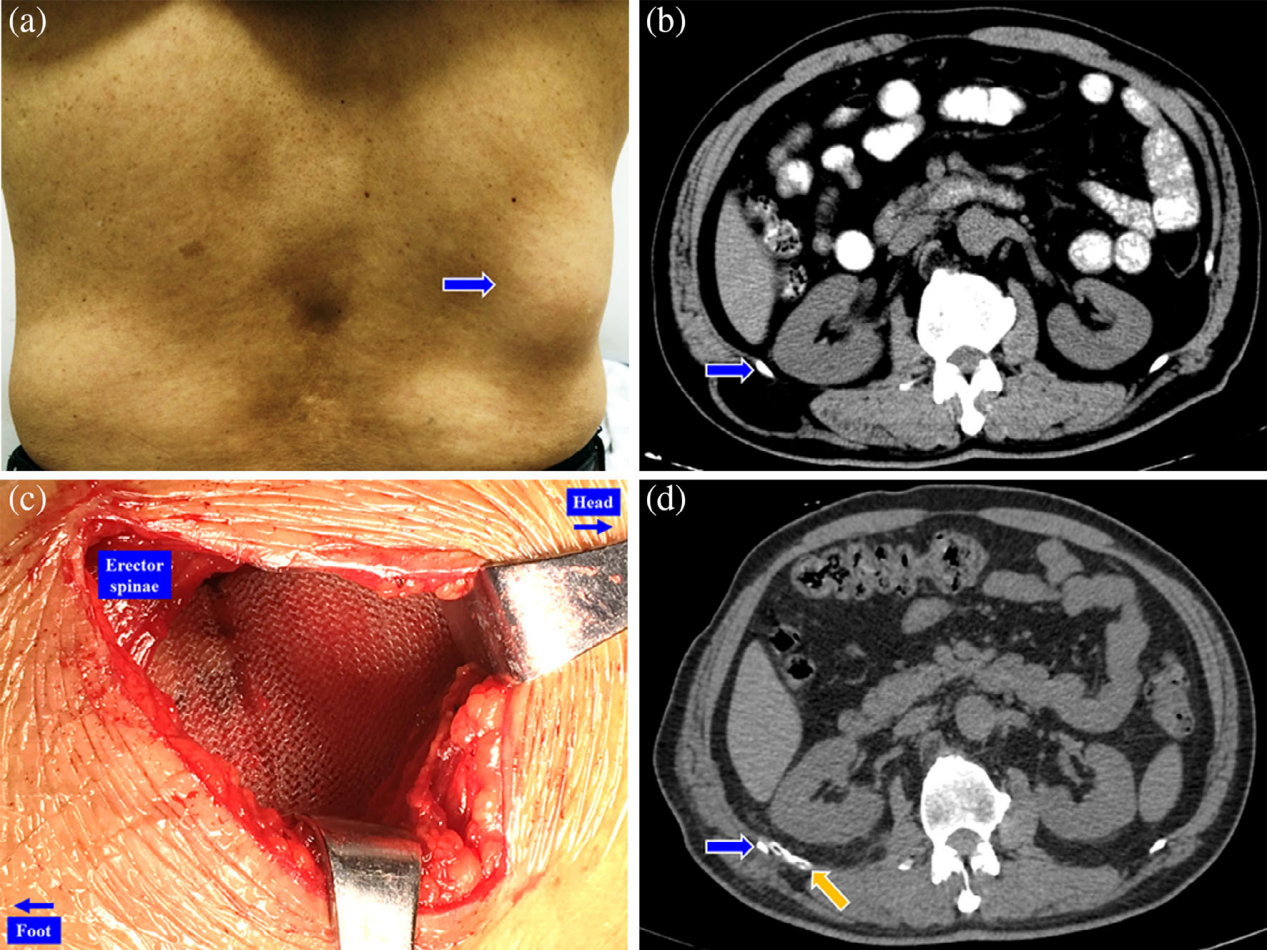
Peri‐operative pictures of a right primary superior lumbar hernia. (a) A physical examination of a right primary superior lumbar hernia (blue arrow). (b) Preoperative computed tomography (CT) scan of a right primary superior lumbar hernia, the blue arrow indicates the 12th rib. (c) Placement of the Kugel patch in the retroperitoneal space. (d) Post‐operative CT scan of a right primary superior lumbar hernia, the blue arrow indicates the 12th rib, while the orange arrow indicates the drainage tube.

Twelve patients with primary superior lumbar hernia who underwent sublay repair with the Kugel patch were enrolled in this retrospective study between December 2008 and June 2019. All patients manifested a characteristic unilateral mass in the superior lumbar triangle (as shown in Fig. 1a), while two patients had simultaneous complaints of constipation. The diagnosis was confirmed by typical manifestations, physical examination and imageological examination, including ultrasonography and computed tomography (Fig. 1b). The informed consents from the patients have been obtained.

### Operative technique

The operation was performed under regional or general anaesthesia, as determined by the anaesthetist. The patients were placed in a lateral decubitus position to provide a better view of the lumbar region. A 5–6 cm oblique incision beneath the 12th rib was performed. The skin and superficial fascia were incised to expose the hernia sac and hernia orifice, and the former was not routinely opened. Adhesion between the hernia sac and hernia orifice was carefully separated to avoid injury to the retroperitoneal organs, including the kidneys and colon. A pocket‐like retroperitoneal space was created by blunt separation with the fingers and the wet gauze technique. An appropriate Kugel patch was placed into the space and was unfolded to cover the defect area (Fig. 1c). The edges of the Kugel patch reached the 12th rib superiorly and exceeded the borders of the hernia orifice by at least 3 cm in the other directions. The hernia orifice was not routinely closed but was fixed to the front layer of the Kugel patch. A closed negative pressure drainage tube was routinely placed in front of the mesh (Fig. 1d) and was removed when the drainage was less than 10 mL. Pressure dressing was routinely applied after the closure of the incision. One patient underwent an ipsilateral inguinal indirect hernia repair with the Kugel procedure, while another patient received an ipsilateral lumbar cyst resection during the operation.

### Mesh

Two different sizes of Kugel patches (Bard; Davol, Inc., Warwick, RI, USA) were used in the study: a medium size of 11 cm × 14 cm and a large size of 14 cm × 17 cm.

### Peri‐operative evaluation and post‐operative follow‐up

Demographic data, including age, gender, body mass index (kg/m^2^), hernia localization, comorbidities and American Society of Anesthesiologists score were collected. The peri‐operative parameters included the hernia defect area, hernia contents, mesh size, operative time, blood loss, visual analogue scale score on post‐operative day 1, removal of drainage, post‐operative duration of hospital stay and days to return to normal activity. For the visual analogue scale score, 0 indicated no pain, while 10 indicated the worst possible pain. The post‐operative data included seroma, haematoma, wound or mesh infection, recurrence and chronic pain. The post‐operative follow‐up was performed through the outpatient service at 1 week, 3 months, 12 months and every 1–2 years thereafter. Regular physical examination and ultrasonography or computed tomography, when necessary, were performed to exclude post‐operative complications. Follow‐up was performed until 30 June 2019, with a median follow‐up time of 51 months (range 3–138 months). The demographic data, peri‐operative parameters and post‐operative data of all patients were prospectively documented in the computer database.

## Results

The demographic data are shown in Table 1, and the peri‐operative parameters are shown in Table 2. Twelve patients with superior lumbar hernias were enrolled in the study, including four male patients and eight female patients. The median age of the patients was 62 years (range from 53 to 72), and the median body mass index of the patients was 24.8 kg/m^2^ (range from 23.1 to 25.9). Six patients had hernias located on the left side, while six patients had hernias located on the right side. The comorbidities included benign prostatic hyperplasia in two patients, chronic cough in one patient and chronic constipation in two patients.

**Table 1:** Demographic data

Demographic data	Patients enrolled (*n* = 12)
Age (years)	62 (53–72)
Gender	
Male	4
Female	8
BMI (kg/m^2^)	24.8 (23.1–25.9)
Hernia localization	
Left	6
Right	6
Comorbidities	
Benign prostatic hyperplasia	2
Chronic cough	1
Chronic constipation	2
ASA score	
I	8
II	4

ASA, American Society of Anesthesiologists; BMI, body mass index.

**Table 2:** Peri‐operative data

Peri‐operative data	Patients enrolled (*n* = 12)
Hernia defect area (cm^2^)	16 (9–25)
5–15	5
>15	7
Hernia contents	
Colon	2
Retroperitoneal fat	10
Kugel Mesh size	
Medium (11 × 14 cm)	5
Large (14 × 17 cm)	7
Operative time (min)	60 (50–80)
Blood loss (mL)	35 (25–50)
VAS/POD1	3 (2–4)
Removal of drainage (days)	3 (2–5)
Post‐operative hospital stay (days)	3 (2–5)

POD1, post‐operative day 1; VAS, visual analogue scale.

All patients underwent an uneventful operation. The median operative time was 60 min (range from 50 to 80), and the median blood loss was 35 mL (range from 25 to 50). The median hernia defect area was 16 cm^2^ (range from 9 to 25). Five medium‐sized Kugel patches (11 cm × 14 cm) and seven large‐sized Kugel patches (14 cm × 17 cm) were used for the repairs. The median visual analogue scale score on post‐operative day 1 was 3 (range from 2 to 4). The median time to removal of drainage was 3 days (range from 2 to 5). Early ambulation was encouraged, and a semi‐liquid diet was then restored 6 h after the operation. The pressure dressing was routinely adapted for at least 3 months post‐operatively. The median duration of the hospital stay was 3 days (range from 2 to 5). All patients returned to normal activities in 2 weeks. No serious post‐operative complications, including seroma, haematoma, incision or mesh infection, recurrence and chronic pain, occurred during the follow‐up period.

## Discussion

Surgical repair is the only method for the treatment of primary superior lumbar hernia. However, no standard procedure for superior lumbar hernia repair exists due to the extremely low morbidity. Tissue repair approximates the muscular layers to close the defect, resulting in high tension, severe pain and a high recurrence rate.^14^ Laparoscopic repairs, including transabdominal preperitoneal repair (TAPP), totally extraperitoneal repair (TEP) and transabdominal partial extraperitoneal repair, have been reported in many studies of lumbar hernia repair. As reported, laparoscopic repair is associated with technical difficulty, and it increased the risks of intra‐operative and post‐operative complications, including organ injuries, chronic pain due to transfascial and tack fixation, and mesh‐related complications such as using a composite mesh.^3^ TAPP and TEP have been applied to small primary lumbar hernias due to the restriction in separating the retroperitoneal space limited by the 12th rib and iliac crest.^15^, ^16^ As compared to TAPP and TEP, transabdominal partial extraperitoneal repair has a wider indication but requires an extensive separation with an expensive mesh and is technically difficult.^17^

Open tension‐free repair seems to be an easy and feasible method for superior lumbar hernia repair, including the onlay, sublay and ‘sandwich’ (onlay + sublay) techniques. Onlay repair has been reported to be associated with the highest recurrence rate and is usually employed as a supplemental repair process for lumbar hernia repair. Sublay repair is an ideal technique for ventral hernias which has been widely accepted^18^ and is also suitable for lumbar hernia repair. Both the sublay and ‘sandwich’ techniques have been reported for lumbar hernia repair in many documents.^19^ However, the meshes used in these studies were common polypropylene meshes with low rigidity and required many sutures for mesh fixation, which remarkably increased the risk of nerve injury. Meanwhile, for ‘sandwich’ techniques, the plane in front of the muscle is to be separated for the onlay mesh which increased the risk of skin flap necrosis. While in the present study, we used the Kugel patch with the sublay technique to repair superior lumbar hernias, which achieved similar effects to those of laparoscopic repair and showed the superior characteristics of a rapid recovery, few complications and decreased difficulty. Meanwhile, this technique is more cost‐effective and easier to perform, with no pneumoperitoneum, no general anaesthesia demand and no chronic pain caused by tacker fixation.

Compared to common polypropylene meshes, the Kugel patch has several unique design characteristics. First, the elastic memory ring helps to unfold the mesh in the retroperitoneal space, achieving a ‘self‐expanding’ effect and effectively preventing mesh shrinking.^20^ Second, the double layers of the polypropylene mesh provide sufficient intensity for lumbar hernia repair, eliminating the need for closure of the hernia orifice. Meanwhile, the double layers of the Kugel patch unfold in the retroperitoneal space and produce an effect of ‘dual’ sublay repair, which is more reliable than that of onlay + sublay repair and with no necessity to create another plane decreasing the risks of would complication. Third, the ‘v‐shaped’ cut and the tissue apposition hole increases the friction between the mesh and surrounding tissues and facilitates the ingrowth of tissues, resulting in rapid fixation of the mesh. Anterior positioning pocket is helpful in mesh placement. As a brief conclusion, the Kugel patch is an ideal material for the sublay repair technique, which requires a sutureless fixation and provides sufficient intensity to strengthen the abdominal wall for superior lumbar hernia. Many surgeons doubted whether the mesh was securely fixed by mere intra‐abdominal pressure^21^ and adapted sutures, tackers or bone anchor fixation for lumbar hernia.^22^ Whereas, no chronic pain or displacement of the Kugel patch, as confirmed by ultrasonography, occurred during the follow‐up period, indicating the safety of the technique.

To date, many controversies still exist regarding the indication for tension‐free repair due to the lack of a standard classification and guideline for lumbar hernias. Tissue repair is recommended when the diameter of defect area is smaller than 4 cm in the China guideline for ventral hernias.^23^ While mesh repair is recommended for all ventral hernia when the diameter of defect area is larger than 1 cm in the updates of guidelines by the international endohernia society.^24^ Regarding to the specificity of superior lumbar hernias, we strongly recommend extending the indication for tension‐free repair. A superior lumbar hernia is a complicated marginal ventral hernia in which the 12th rib is the superior border. Meanwhile, the directions of the three boundary muscles are notably different from each other, and as a result, the muscles are difficult to approximate. Accordingly, the defect area is difficult to close completely, leading to a high recurrence rate of tissue repair.^25^ In addition, a superior lumbar hernia is often diagnosed with a delay due to its concealed manifestation, resulting in an enlarged hernia orifice during treatment. According to Loukas's classification,^26^ lumbar hernias are classified into four types based on the size of the defect area: type I < 5 cm^2^, type II 5–15 cm^2^, type III >15 cm^2^ and type 0 no triangle is formed. In our study, five patients had type II hernias that were repaired with a medium‐sized Kugel patch, while seven patients had type III hernias that were repaired with a large‐sized Kugel patch. For ventral hernia with larger defect areas, mesh reinforcement repair with closure of hernia orifice is recommended in the international endohernia society guideline.^24^ Whereas, due to the difficulties in closing the hernia orifice, bridge repair is often adapted in superior lumbar hernia repair. However, sublay repair using the Kugel patch could provide sufficient reinforcement with double‐layer mesh even with only bridge repair. No recurrences occurred in the present study, which demonstrates the reliability of this technique.

The main limitation of the study is that this is a retrospective clinical study with only a few patients enrolled instead of a randomized controlled trial with a large case–control study. However, the small sample size is mainly attributed to the extremely low morbidity of lumbar hernias. A study with a larger sample size is required in the future to further confirm the effectiveness of this technique.

## Conclusion

In conclusion, sublay repair for superior lumbar hernia using the Kugel patch is a feasible, cost‐effective, safe and effective alternative method for superior lumbar hernia repair. This technique shows the benefits of minimal invasiveness and a rapid recovery, and is easy to learn and perform.

## Conflicts of interest

None declared.
